# “Souls of the ancestor that knock us out” and other tales. A qualitative study to identify demand-side factors influencing malaria case management in Cambodia

**DOI:** 10.1186/1475-2875-11-335

**Published:** 2012-10-05

**Authors:** Kathryn A O’Connell, Ghazaleh Samandari, Sochea Phok, Mean Phou, Lek Dysoley, Shunmay Yeung, Henrietta Allen, Megan Littrell

**Affiliations:** 1 Department of Malaria and Child Survival, Population Services International, P.O. Box 14355-00800, Nairobi, Kenya; 2 Department of Maternal and Child Health, University of North Carolina at Chapel Hill, Gillings School of Global Public Health, Chapel Hill, NC, USA; 3 Population Services International Cambodia, No. 29 Street 334, BKK1 Chamcar Mon, P.O. Box 153, Phnom Penh, Kingdom of Cambodia; 4 National Centre of Entomology, Parasitological and Malaria Control and School of Public Health, National Institute of Public Health, House 372, St Monivong Vong, Boeung Keng Kang I, Chamcar Mon, Phnom Penh, Kingdom of Cambodia; 5 Department of Global Health and Development, Malaria Centre, London School of Hygiene and Tropical Medicine, 15-17 Tavistock Place, London, WC1H 9SH, UK

**Keywords:** Treatment-seeking behaviour, Patient perceptions, Patient-provider interactions, Malaria diagnosis, Malaria treatment, Cocktail, ACT, Cambodia, Qualitative research

## Abstract

**Background:**

Appropriate case management of suspected malaria in Cambodia is critical given anti-malarial drug resistance in the region. Improving diagnosis and the use of recommended malarial treatments is a challenge in Cambodia where self-treatment and usage of drug cocktails is widespread, a notable difference from malaria treatment seeking in other countries. This qualitative study adds to the limited evidence base on Cambodian practices, aiming to understand the demand-side factors influencing treatment-seeking behaviour, including the types of home treatments, perceptions of cocktail medicines and reasons for diagnostic testing. The findings may help guide intervention design.

**Methods:**

The study used in-depth interviews (IDIs) (N = 16) and focus group discussions (FGDs) (N = 12) with Cambodian adults from malaria-endemic areas who had experienced malaria fever in the previous two weeks. Data were analysed using NVivo software.

**Results:**

Findings suggest that Cambodians initially treat suspected malaria at home with home remedies and traditional medicines. When seeking treatment outside the home, respondents frequently reported receiving a cocktail of medicines from trusted providers. Cocktails are perceived as less expensive and more effective than full-course, pre-packaged medicines. Barriers to diagnostic testing include a belief in the ability to self-diagnose based on symptoms, cost and reliance on providers to recommend a test. Factors that facilitate testing include recommendation by trusted providers and a belief that anti-malarial treatment for illnesses other than malaria can be harmful.

**Conclusions:**

Treatment-seeking behaviour for malaria in Cambodia is complex, driven by cultural norms, practicalities and episode-related factors. Effective malaria treatment programmes will benefit from interventions and communication materials that leverage these demand-side factors, promoting prompt visits to facilities for suspected malaria and challenging patients’ misconceptions about the effectiveness of cocktails. Given the importance of the patient-provider interaction and the pivotal role that providers play in ensuring the delivery of appropriate malaria care, future research and interventions should also focus on the supply side factors influencing provider behaviour.

## Background

In Cambodia, an estimated 2.65 million people are at risk of malaria [1]. The Cambodian Ministry of Health estimates that 83,777 outpatient and 4,045 inpatient malaria cases were reported in 2009, with this disease accounting for 0.6% of all outpatient cases and 3.5% of all inpatient cases in the same year [2]. Estimated prevalence rates range from 3.0% to 12.3% in malaria-prone provinces, with the epidemiology of malaria varying widely across the country. Prevalence is highest around the tropical forests located on the country borders, covering 60% of Cambodia’s landmass. Parasite prevalence rates vary and are reported to reach 15% to 40% in remote, forested areas, with much lower rates in the plains [3]. In the northeast, malaria transmission is relatively high; the reported annual incidence rate lies between 11 to 50 cases per 1,000 habitants and *Plasmodium falciparum,* the deadliest strain of malaria, predominates [4]. By contrast, along the western border with Thailand, *P. falciparum* malaria transmission is generally lower than the northeast and *Plasmodium vivax* predominates [5,6]. Malaria transmission risk in Cambodia is associated with the rainy season, typically peaking in August and September. Unlike many areas of sub-Saharan Africa, the highest burden of malaria infection afflicts adults who work and stay overnight in the forests.

The border between Cambodia and Thailand serves as an epicentre of multidrug resistance [5,7]. Since the 1970s, this area has been the hotspot for the development of anti-malarial resistant parasites; resistance to anti-malarials, including chloroquine and sulphadoxine-pyrimethamine, subsequently spread to other parts of Asia and Africa [8]. In 2009, artemisinin-resistant *P. falciparum* malaria was confirmed in Cambodia’s Pailin province [9]. Experts believe a number of factors have contributed to the emergence of drug resistance in Cambodia: 1) previously unregulated sales of artemisinin monotherapy; 2) limited access to artemisinin combination therapy (ACT); 3) ACT that are not co-formulated (facilitating continued use of artemisinin monotherapy); and 4) ubiquitous counterfeit and substandard medicines [9].

Over the past 10 years, the Cambodia National Malaria (CNM) programme has pioneered a number of innovative malaria control approaches, many of which have become accepted as standard practice in malaria-endemic nations. For example, since 2000, CNM has recommended using ACT (artesunate and mefloquine) as the first-line treatment for *P. falciparum* malaria and chloroquine as the first-line treatment for *P. vivax* malaria. Before treatment, the National Treatment Guidelines instruct providers to confirm malaria infection through microscopy or a rapid diagnostic test (RDT). In 2008, CNM changed the protocol for treating malaria in districts with confirmed multidrug resistance, switching to dihydroartemisinin + piperaquine (DHA + PPQ), a fixed-dose combination, as the first-line treatment. Under the Resistance Containment Programme, CNM launched multiple initiatives including a ban on the sale of artemisinin monotherapy [10] as well as community level services to facilitate rapid diagnosis and treatment with the correct first-line anti-malarials [11]. Other national malaria control efforts include the provision of highly subsidized RDTs and ACT treatments in the private sector since 2003, the provision of these commodities for free in the public health sector [12,13], and regular monitoring of the quality of anti-malarials in both the public and private health sectors at sentinel sites [14].

Despite these efforts, recent research in Cambodia shows that rates of diagnostic testing and prescription of first-line treatment for confirmed cases remains relatively low among persons with malaria fever. Supply side data from outlet surveys show that the availability of diagnostic tests and the first-line treatment is variable, with higher availability in the public sector, but lower stocking rates in the private sector [15]. As such, when patients seek out treatment for malaria fever, the diagnostic tests and/or the first-line treatment may not be available. In addition, other supply side research has shown that many of the anti-malarials may be sub-standard or fake in Cambodia [14]. Moreover, providers may prescribe unsuitable dosages, incorrect medicines and improper duration of treatment [16]. In addition, household survey data suggest that many people with malaria fever rely on home remedies, such as sponge baths and traditional medicines made from a variety of herbal or plant sources, which they self-administer [17,18]. This reliance on self-treatment with home remedies may delay patients from seeking proper care. In addition, while nearly half of all Cambodians who seek care for malaria symptoms receive a blood test, patients most commonly received medicines sold or dispensed by health providers as “drug cocktails” when treating these fevers [18], a finding supported by other quantitative research [11,19]. Cocktails typically consist of a small plastic bag containing one or more tablets of various medicines including antipyretics, vitamins, anti-malarials, antihistamines and antibiotics [11].

The widespread use of cocktails creates challenges and dangers for combating malaria in Cambodia. First, as it is the provider who decides on the composition of the cocktail, it is unclear what patients receive in their plastic bags and whether they even receive an anti-malarial. If an anti-malarial is provided, it may be an incomplete dose or an oral artemisinin monotherapy – both of which lead to parasite drug resistance [20]. The variation in the number of cocktail packets bought from the provider adds another threat to combating drug resistance. Even though providers often present multiple packets or pills as a full course of treatment, some patients do not always choose to purchase a full course; factors such as affordability and illness severity sometimes limit the number of cocktail packets that patients buy [11]. For these reasons, Cambodian national malaria control efforts have also focused on increasing consumer awareness of the dangers of cocktail medicines through behaviour change communication (BCC) campaigns.

Efforts to change how Cambodians approach malaria treatment face notable challenges. In general, treatment-seeking behaviour for illness is a highly complex process. Around the globe, people frequently seek multiple sources of treatment and many self-medicate or undergo some type of treatment at home, outside of a medical facility. People also often have specific perceptions of medicines, believing that some are more effective than others. Moreover, specific cultural beliefs, norms and attitudes are likely to influence the treatment-seeking process [21-24]. Numerous research studies, primarily conducted in sub-Saharan Africa, have extensively documented a number of demand-side factors associated with treatment-seeking behaviour, including perceptions about the cause and severity of the illness, quality of care at health facilities, affordability of treatment, proximity of services to patients, and positive manner of the providers [21-25].

In Cambodia, the epidemiology of malaria and the specifics of the treatment environment make the malaria treatment-seeking process vastly different than that found in other parts of the globe, particularly the process in sub-Saharan Africa where most research on this topic has focused thus far. Cambodian adults – specifically forest workers – are most afflicted by malaria, unlike in much of Africa where children under five are most at risk. As a result, caretaking responsibility rests with the individual rather than the caregiver of a young child and access to care is limited, factors which often guide treatment-seeking decisions in Cambodia. Moreover, while self-treatment of fever at home is common worldwide, the practice in Cambodia appears to be much higher than in surveyed sub-Saharan Africa countries [26]. Cocktail medicines are also more commonly used in Cambodia and the Southeast Asia region [11,20], a negligible practice in sub-Saharan Africa [26]. Finally, malaria treatment practices in Cambodia are complicated by the multiple definitions and cultural understandings of “fever”. Cambodia’s main language, Khmer, uses a variety of terms and definitions for fever such as: fever with chills (*krun janh*) or hot body (*krun ngak*) known as “malaria fever”; dengue fever (*krun chhiem*); or other types of fever or symptoms, such as night fever (*krun yop*), high temperature (*kdao gadow/kdao kluan*) or sweating (*krung loap)*[27]. Such variety may further complicate treatment practices.

To date, only a few unpublished papers provide a descriptive picture of the factors that tend to influence malaria treatment in Cambodia [28,29]. One of these studies, a qualitative study from 2004, suggests that a number of factors influence provider decisions in Cambodia [28]. These include stock outs of test kits and financial incentives to sell medicines rather than test before providing treatment, since a confirmed diagnosis may diminish medicine sales due to negative test results. Results also revealed that patients may prefer to spend money on medicines rather than a test, and may prefer to self-treat based on their symptoms until they become more seriously ill [28].

To expand the limited evidence base, this study uses qualitative methods to explore the demand-side factors that influence malaria treatment-seeking behaviours and patient-provider interactions among Cambodian patients. It aims to shed light on findings from quantitative research studies and offer programmatic recommendations to increase the uptake of appropriate malaria case management in Cambodia. It asks three key questions: 1) Why do people first treat at home and what types of medicines are used? 2) Why do patients take drug cocktails for malaria and what are their perceptions of these medicines? and 3) Why do some patients with malaria fever receive a diagnostic test while others do not? The study findings aim to provide a more nuanced understanding of the patient-provider interaction at a health facility or outlet where malaria treatment is sought, including a patient’s perception of the provider. Such findings may prove useful in guiding the design of interventions focused on increasing informed demand for effective malaria case management services in Cambodia.

## Methods

Researchers employed qualitative in-depth interviews (IDIs) and focus group discussions (FGDs) to investigate treatment-seeking behaviours for malaria fever in Cambodia. To understand the complexity of factors related to diagnosis and treatment-seeking behaviours, two groups of participants were recruited: adults who reported they had their malaria fever confirmed through diagnostic testing and those who reported they did not confirm their fever with a diagnostic test. FGDs gathered information on community level norms and beliefs about parasitological diagnosis and malaria treatment-seeking behaviours. IDIs collected data on participants’ individual experiences when they fell ill with fever, their treatment-seeking processes and the dynamics of the patient-provider interaction when seeking care for malaria.

### Sampling

A non-probability, purposive sample of the target group was used to recruit study participants from three randomly selected, rural, malaria-endemic districts, located in the heavily forested areas of Pursat and Kratie provinces. To find participants, researchers employed a variety of snowball sampling methods. First, the village chiefs, health providers, and shop assistants and owners who sell medicines from various outlets (e.g. pharmacies, drug stores, etc.) served as key informants to identify people in the area who recently had fever. Potential participants were then asked if they knew of other people in surrounding villages or forest areas who had experienced malaria fever (*krun janh/krun ngak)* in the previous two weeks.

Researchers used a screening questionnaire to determine the respondent’s eligibility for inclusion in the study. They asked whether or not the respondent had had malaria fever (*krun janh or krun ngak*) in the previous two weeks, as opposed to other common types of fever in Cambodia, such as dengue fever (*krun chhiem)* or night fever (*krun yop*). They also ascertained whether or not the potential participant’s malaria fever had been confirmed using a diagnostic test.

Aiming to conduct at least 10 FGDs, with 8-10 participants each, and 12 IDIs, the research team implemented 12 FGDs and 16 IDIs over a two-week period during the rainy season in August 2009. Researchers sought to enrol a similar number of participants into each of the two sampling groups, those who had received a diagnostic test and those who had not.

### Data collection

Teams of four Cambodian social scientists (two women and two men) hired from the local community conducted the IDIs and FGDs in Kratie and Pursat provinces. All were trained to correctly use the guides and study protocols for data management. They held each in-depth interview in a private space, sometimes at the home of the individual. FGDs were held in community centres. Prior to participation, researchers informed study participants of the study objectives and obtained verbal consent from all participants. Incentives were provided in the form of refreshments after participation as well as a traditional Khmer scarf (*kroma*). Researchers made voice recordings of all IDIs and FGDs, with the consent of participants, and interviewers also took notes of the content, non-verbal behaviour and setting of the interaction.

Interview and focus group guides with open-ended questions addressed key topics related to treatment-seeking behaviour. Each of these instruments focused on *how* participants responded to their fever, *where* they sought treatment, *why* they did or did not receive a diagnostic test, *what types* of treatment they received and the *perceived efficacy* of those treatments. While both the IDI and FGD instruments asked respondents to describe their most recent episodes of fever, FGD participants discussed their recent fever experiences within the group as a means for helping understand the cultural and social norms around malaria treatment-seeking behaviour. The IDI and FGD guides were translated, piloted and revised before and during the fieldwork. This process aimed to improve the clarity of the questions, assist in assessing topic saturation to guide any needed changes in the instruments, and allow for any required increases in the sample size.

### Ethics

Ethical approval was obtained for this study from the Cambodian Ministry of Health Ethical Review Board, 19 July 2009 (#109NECHR).

### Data analysis

Recordings from all interviews were transcribed verbatim in the original Khmer and then translated into English. On completion, a member of the bilingual research team read and reviewed each translated English transcript, comparing it against the original Khmer version. The team rectified any discrepancies with the translator until full agreement between the translated transcript and original Khmer version was obtained.

Researchers used NVivo qualitative data analysis package (QSR International Pty 2002) to analyse the data. Following the principles of grounded theory, the research team coded the transcripts according to common themes that emerged from the data, letting the data guide the coding rather than allowing researchers to impose a coding scheme [30]. Given the open nature of the interview questions, this approach enabled the emergence of unexpected concepts and categories that sometimes had dual meanings, such as “trust in a provider”. These concepts were inductively generated and coded, meaning that the same respondent could be coded for expressing both trust and lack of trust in a provider. Researchers analysed the frequency with which individuals reported themes, and to what extent the group mentioned these themes, for patterns. This procedure helped clarify which themes consistently emerged across all groups and which were idiosyncratic [31]. To capture the main topics emerging from the data, the research team arranged the descriptive codes into sets of broader themes. For example, the code, “I always believe providers will give me the right treatment to cure my fever” was categorized under the theme, “trust in providers”. Researchers avoided coding data into categories that were too small (e.g. “belief that cocktail medicines will cure fever immediately when working in the forest”), as such classification can make results difficult to interpret [32]. Therefore, the team created codes to encapsulate recurring themes, eventually revisiting the entire dataset with the final coding scheme to perform the final analysis.

To ensure inter-rater reliability in the coding, the research team employed various procedures. First, the Khmer research staff conducted a paper-based, thematic analysis on 40% of the IDI and FGD transcripts, using the final codes that had emerged from the initial analysis phase. Then, they compared the findings between the Khmer transcript-based analysis with the initial findings from the Nvivo analysis. Through this comparison, the team checked to make sure they had coded the themes in a consistent manner, without creating new codes in one type of analysis and not the other. Any discrepancies were resolved through discussion with the larger team of coders and the primary analyst until full agreement was obtained.

To confirm the reliability of the findings, the lead researchers presented the results to the Khmer research team upon completion of the analysis. In addition, the team once again verified any quotes used in the results summary with the translators.

## Results

A total of 60 participants (68% male, 32% female) participated in this research study, with a mean age of 32 years. Most respondents had no secondary education: 75% had received primary school education alone while 15% had finished secondary education. Another 10% had not received any schooling at all. The large majority of participants were married (85%) and reported working in forested areas (80%). Per the sampling criteria, all participants reported having malaria fever in the previous two weeks. 27 participants had received a diagnostic test upon seeking treatment while 33 had not.

It should be noted that the sampling groups – those who sought diagnostic testing versus those who did not – did not have any bearing on the results, with the exception of the expected differences in diagnostic testing. The emergent themes remained consistent within each group, so differences in use of self-treatment and cocktail medicines did not appear.

### Findings

#### Types of treatment taken at home

Few participants reported taking immediate steps to treat their fever. Most waited a few days before seeking treatment outside the home, either because they were waiting for symptoms to worsen and/or to ensure the fever was not the result of a simple cold or flu. They also waited because they were in the forest, far away from health centres or outlets where they could purchase medicines.

Respondents also reported that medicines are expensive and they did not have the money to treat their fevers. Thus, a common alternative is to treat the fever at home, using traditional remedies. Many believe the symptoms can be treated successfully at home, an impression that is also rooted in their perception of the seriousness of the illness.

Moderator: When you have a hot temperature, where do you go for treatment?

Respondent 1: First, we treat it at home by ourselves. We use different medicines to make us feel better, and which help reduce the fever. The elders say to use a kind of roof thatching plant, corn and a kind of aquatic herb that is used as a spice and which keeps for a year. We boil the core of the corn and use this to treat the fever.

Respondent 2: But if the fever is not any better after two or three days, we may go to a hospital or a shop that sells medicines.

Respondent 3: But if it is serious from the start, we will go to the hospital right away.

*FGD, Pursat Province*

Home treatment includes traditional medicines, primarily used to delay, alleviate and/or cure symptoms before seeking modern alternatives. Most reported drinking boiled tree roots (e.g. the roots of the lemon tree with alcohol or kapok leaves) or taking warm sponge baths with fragments of ginger, or with guava leaf and the leaf of a small, sour fruit. They also use other natural remedies, as described by this forest worker:

*Respondent: If we are in the forest, we have nothing with us. So, we just have trees in the forest such as “Ampil Brok Phler” and “Merm Krovanh Chruk”. We just eat them when we do not have medicines. Moreover, there are “Cheung Kras” grass, and also coconut stumps which we cut to eat. These are temporary medicines in the forest until we arrive home. When we have a serious fever, we can cut the coconut core into two pieces, tie it with black thread, insert a nail, and then boil them together to drink until we get medicine. … The* [traditional medicines] *can help around 30% or 40%. They can protect us from running a high temperature. But these won’t be effective for long, so, three or four days later, we will start shivering again. However, this mixture helps us to be able to ride our ox cart home *

*IDI, 32 year-old, Male Forest Worker, Pursat Province*

While treatment at home is commonplace among respondents, they generally sought help outside the home from a qualified provider of health care if the home treatment strategies were perceived as ineffective or failing, or if symptoms worsened. This source outside the home was most often the nearest provider of modern medicines. Sometimes respondents resorted to using modern medicines that they had left over from a previous episode of illness, even if these medicines were not anti-malarials.

#### Reasons for taking cocktails

While many respondents rely on traditional medicines as a primary treatment, the majority of participants eventually receive some form of modern medication for their malaria symptoms. A few participants mentioned brand names without prompting from the interviewer or FGD facilitator, typically the ACT *Malarine* (private sector) or *A + M* (public sector). However, most respondents did not name the medications they received. The most common point shared by all respondents about modern medications focused on cocktails. When they sought modern treatment, they often received medicines without formal packaging, presented as cocktails. Therefore, this section focuses on the study results surrounding the perceptions of cocktail medicines, rather than the perceptions of pre-packaged medicines.

Respondents reported the cocktail medicines are presented in different forms. Sometimes, the cocktails consist of complete blister packs that have simply been removed from pre-packaged medicine boxes. In other cases, providers have cut up the blister packs or removed the pills from the blisters or tins altogether, placing these individual pills in the mixture. The providers prepare all of these formulations in small plastic bags. Instructions for taking these cocktail medicines direct participants to take them multiple times a day over the course of several days, typically using the colour of the pill in the cocktail (e.g. blue, white, yellow or red) to denote which medicines to take at specific times. Sometimes the instructions describe a sign or picture imprinted on the pill(s). Some respondents reported linking the colour of the pill with the perceived curative agent. For example, they described the red pills as the pills for energy, while the light blue pills were “new” treatments for malaria.

Moderator: What sort of medicine were you offered?

Respondent 1: I got pills in a plastic bag. The provider removed different pills from a blister pack and some from a big container, and put them into the plastic bag.

Moderator: The provider removed them from the blister pack?

Respondent 1: Yes, she did.

Moderator: What about others here? What medicine did you get?

Respondent 4: I don’t know. The health provider just prepared a bag for me to take twice per day, morning and afternoon. I didn’t understand [anything] about those medicines. I could not recognize them, but I knew they related to malaria and typhoid. The health providers told me. They gave me the tablets after I got serum.

Moderator: Did the medicine come with its original package or cover?

Respondent 4: Some came with its original packaging, some didn’t. It came in a small plastic bag.

*FGD, Pursat Province*

Among study participants, the use of cocktails is widespread and is viewed as a “normal” medicine given by providers. Given the frequency with which respondents mentioned receiving cocktails, this type of treatment is deemed a common, standard treatment for all symptoms. Respondents did not question the efficacy of the cocktail treatments, regarding them as a “normal” and “common” type of treatment for malaria from health providers. They cited trust in the efficacy of these treatments. In addition, respondents perceive the inclusion of multiple types of pills in cocktail packages as more effective and less expensive than a pre-packaged medicine. Many described the need to not only cure malaria, but also to reduce fever and headaches; thus, a combination of pills, targeting multiple symptoms, is deemed more effective and more affordable than purchasing multiple pre-packaged medicines. Respondents also generally cited trust in their providers and believed that the provider would only give them an effective medicine:

Interviewer: Do you think these [cocktail] treatments are effective?

Respondent: Yes, they are good treatments because I always get better after treating [my illness with them]. And I believe in the medicines because they are given to me by health providers. They save us. It is the provider’s job to save us.

Interviewer: How much do you believe in these treatments?

Respondent: I believe almost 100%.

*IDI, 29 year-old, Male Forest Worker, Kratie Province*

Many respondents also discussed how the cocktails are often saved for later illnesses, or for when they return to the forest. They also reported stopping the medicine regimen prematurely because they felt better, or they needed the medicines for other family members or their own future illnesses, as illustrated by this exchange:

Respondent 2: He [the provider] told me to take it [the medicine] regularly and on time, and that I have to finish it even if I am better. And I did follow his advice.

Moderator: What about others?

Respondent 3: No, I will finish all of it [the medicine].

Respondent 4: I keep one in reserve in case we are sick next time.

Respondent 5: For me, they prepared four to six bags for me. And after I finished four bags, my kid became sick. I knew he had malaria as his toes were cold and he had a headache… and he told me he felt numb. His face was also numb. So I gave him some [medicine]. For older people, we need to take only one tablet, so I cut it into two parts [for my son]. I just give him half a tablet. After I gave him the medicine, he got better.

*FGD, Pursat Province*

#### Reasons for diagnostic testing

The data suggest that many respondents are not aware they need a diagnostic blood test to confirm their fever as malaria. Many also believe they do not need to do a test because they are able to self-diagnose malaria. Since many participants claimed they “knew” it was malaria because the symptoms they had experienced were the same as those from previous bouts of the illness, they felt that the additional cost imposed by testing seemed fruitless. Sometimes this perception was linked to previous experiences with diagnostic testing; those who had had prior experience with tests that confirmed fever as malaria now felt in a better position to recognize when fever episodes were malaria. Others reported that providers did not mention the need for a test. Some participants also noted that they did not know where to get tested.

Respondent 3: For me, I just went to the shop and got the medicines. The provider did not tell me to take a test or anything. He gave me medicines and told me to take them to cure my fever. So I did. I didn’t know anything about a test.

Respondent 2: I just got the medicine, too, from the place near my house. It was given to me for curing my malaria. I don’t even know where I should find such a test for malaria.

Respondent 5: Going to have our blood tested once is enough for us. Next time if we have the same symptoms, we do not have go get diagnosed again. Instead, we can just buy medicine from the drugstore. We know when it is really malaria.

Respondent 8: From my experience, I am used to having malaria. I observe my son and myself [when we get sick]. It starts first with yellow eyes. The eyes start being yellow after we return from the forest. When I open his eyes, it looks like there is not any red blood inside them. It’s all a big fever virus. Some called it like the souls of our ancestors knock us out. It starts with feeling cold and hot temperatures again and again. No chills, but after three days, it becomes a serious fever, and then you become unconscious. From this, I know it is malaria.

*FGD, Kratie Province*

For respondents who mentioned seeking treatment from public or private facilities or clinics, many did not question the need for a diagnostic test because they trusted their provider to give them the appropriate treatment. This finding contrasted with responses from those who obtained treatment from less formal outlets including village shops or markets; these respondents did not comment on the quality of the provider. Participants perceive public and private health care providers as knowledgeable and experienced, as well as able to ascertain what type[s] of medicine is needed based on their symptoms. Providers are viewed as being a source of authority in treatment and diagnosis. Several respondents reported they would accept any decision handed down by the provider. Often they were not challenged by the provider to have a test, as illustrated by the following discussion:

Respondent: When I got to the health facility, I told the provider to give me anti-malarial medicine. Also, the provider did not request or provide blood testing for me, but only gave me a cocktail.

Interviewer: So what did you tell the provider?

Respondent: When I arrived, I asked him if he could please give me anti-malarial medicine “for three times” and he did that for me. In fact, he also has test equipment [but he didn’t use it on me].

Interviewer: Why did you ask him to give you anti-malarial medicine?

Respondent: Because I think my disease is really malaria. So I just tell him to give me anti-malarials, which he did for me.

Interviewer: So, he did not ask you anything?

Respondent: He did ask me a few questions, something like “How did you get malaria?”

*IDI, 22 year-old Female, Kratie Province*

In contrast, those who received diagnostic testing noted that blood tests are also seen as part of the treatment plan, as well as something that is prescribed by providers. The data suggest that receiving a test is often dependent on where people purchased or obtained medicines. Typically, respondents mentioned receiving a test when they sought treatment from a public health facility or a private hospital. They also talked about the role of the provider in this process, citing that receiving a test is up to the health provider, either public or private, as illustrated by the quote below:

Respondent 5. When I arrived, the doctor said that I have to have a blood test, in order to make it easier to prescribe the proper treatment. If we do not have a blood diagnosis, we cannot know what disease it is, and so we cannot provide proper treatment. After the blood test, I was informed I have three positive signs of malaria and I was given A + M.

Respondent 7: First, my feet were cold and I felt my neck was cold too. So, then I warmed myself by the fire. After doing this, I was still cold. Then, I went to sleep without taking any medicine. I started out really cold until I covered myself with a blanket, but I was still cold. So, I took two tablets of paracetamol, which [made me feel] better, although I was sweaty for a while. Then the chills started again. After that, I was brought to the hospital.

Moderator: How many days were you sick before you went to the hospital?

Respondent 7: Three days, then I went to the hospital. As I did not recover, I went to the hospital for a blood test. They asked me where I had been. And I said that I had been in the highland area. Then they asked me to have a blood test. So, I took it.

*FGD, Kratie Province*

In other instances, participants said they specifically sought a diagnostic test in order to understand their illness, or in order to correctly diagnose the illness and find the appropriate treatment. In these cases, although they had familiarity with some malaria symptoms, they recognized they could not identify the illness on their own and, therefore, needed a blood test to confirm the cause of the fever. Others reported taking medicine first without being tested. When the symptoms did not improve, they decided testing was necessary to find the “right” treatment. In addition to aiding the identification of the correct treatment, confirmation of disease also prevented the risky behaviour of taking an anti-malarial when it was not needed. Doing so was seen as “dangerous”. Participants explained the effects of unnecessary anti-malarial treatments as “harming the blood vessels”, “making blood thick”, “weakening the blood” or “shattering the blood bullet”.

Respondent: Because I felt uneasy inside my body, I even took traditional medicine as well as the cocktail which I had bought nearby. I did not recover. I just spent money on the medicine without getting good results. That was why I needed the blood test.

Interviewer: When you got the blood test, did you request the provider to do it for you or did you just go there to see what the provider would recommend?”

Respondent: I asked him to do it because I felt strange in my body. I always had chills and fever and could not get better by taking the medicine. And I wanted to know what illness it was, and why I did not get better even after taking the medicine. So, I asked him to do the blood test to identify the disease.

Interviewer: Before you did the blood test, what did you take?

Respondent: Beforehand I just took medicine like paracetamol and stuff like that for when you have a fever, and also the thing called Tetra [tetracycline].

Interviewer: Oh, so you didn’t have anti-malarial medicine?

Respondent: No, that time I did not know I had caught malaria until I did the blood test. If I had known that I had caught malaria, I could have chosen the right medicine

*IDI, 34 year-old, Male Forest Worker, Kratie Province*

Respondents also reported taking a blood test before going to work in the forest, particularly if they were experiencing malaria symptoms already. They wanted to ensure the malaria was cured before they travelled long distances where they would be required to work and sleep in areas far from any health facilities. They also reported taking cocktails with them in order to have medicine on hand in the forest in case they experienced new symptoms, as this participant demonstrates:

Interviewer: Uncle, did you do the blood test?

Respondent: I did it before going to the forest.

Interviewer: How many days did you do the blood test before you went to the forest?

Respondent: Three days before I went to the forest. However, before I went to the forest, I already had had fever one or two times. That is why I did the blood test. I thought I could not take cocktails if I go to the forest again and I do not know what disease I catch. So I needed to do the blood test to see what illness I have. Then, I could buy cocktails to take to the forest.

*IDI, 45 year-old, Male Forest Worker, Pursat Province*

A number of respondents talked about obtaining multiple blood tests. They reported that providers could not recognize the results or needed to double-check the results at times. Many participants also reported confusion in ascertaining what the results of their tests were. Sometimes, they were unclear if they had tested positive for malaria or other illnesses, namely dengue fever or typhoid. In some cases, providers had given respondents anti-malarials even though their test results were negative. Others reported getting mixed results from the test:

Respondent 7: First, I had a blood test. The results showed that I was positive for malaria. I recovered after I took medicine for malaria. Then I got the chills again at 8 o’clock. I told my mother that I was not recovered yet. So, my mother took me to have a blood test again. The results showed I was positive for typhoid, not malaria.

*FGD, Pursat Province*

## Discussion

The qualitative results presented in this paper shed light on the complexity of treatment-seeking behaviour for malaria among Cambodians, an important issue to understand in order to effectively implement appropriate malaria case management in the country. This study provides a richer understanding of how patient decisions about treatment choice, the sequence of treatments (from home-based to facility-based care) and diagnosis are based on multiple factors. These factors include prior fevers, treatment experiences, local beliefs about how fever should be treated, the influence of social networks, practical considerations such as cost and proximity to health facilities, and cultural norms. This section discusses these issues in more detail and suggests interventions that may improve fever case management and treatment-seeking behaviour among Cambodian patients.

Cambodian cultural practices, norms and beliefs, as well as practicalities and malaria episode-related factors, drive decision making about treatment practices. As demonstrated by other research studies [18,26,33,34], this study shows that treatment-seeking behaviour for malaria in Cambodia often starts with self-treatment prior to any biological diagnosis. When deciding how to treat oneself for suspected cases of malaria fever, Cambodians who work in forested areas weigh their beliefs in the effectiveness of these home-based treatments against their appraisal of available outside treatment options, such as proximity to health facilities, availability of financial resources and perceptions of the illness’ severity. Others have also found that the perceived severity and duration of illness are influential in the decision to seek and obtain treatment [21,22,24]. The Cambodians examined in this study generally seek health care outside the home when the self-treatment strategies are perceived as failing, or when the illness is perceived as worsening. However, even if they do intend to obtain a confirmed diagnosis and/or obtain appropriate treatment, making the decision to seek outside care is impacted by transport costs, care costs, the distance to a drug outlet and knowledge of tests, as confirmed by others [21,22,24]. Treatment-seeking behaviour is also mediated by previous treatment-seeking behaviour. As evidenced by other research [35], leftover medicines from previous illness episodes are reportedly saved and administered when another family member becomes ill.

Cambodian social norms also encourage the use of drug cocktails for malaria treatment. Cocktail use is widespread, a finding demonstrated by this study and confirmed by other quantitative [11,14,16,17,27] and qualitative [28] studies in the country. This study’s interviews and focus groups showed that cocktails are the accepted community norm to treat illnesses, suggesting that this form of medicine is provided for other types of illness, not just malaria. The perceived affordability of these cocktails, compared to pre-packaged therapies, is a strong driver of this community norm. Drug cocktails are believed to treat multiple symptoms more effectively. Other evidence has demonstrated similar conclusions, noting that cocktails are preferred because they are less expensive than pre-packaged treatments and patients can only afford to pay for a certain number of pills at one time [28].

This study also unveiled the importance of the patient-provider interaction – and the cultural norms influencing this interaction – as pivotal in the context of malaria diagnosis and case management in Cambodia. Because providers are perceived as providing sound advice and knowing the best or correct treatments for malaria, respondents noted that they readily accepted the provider’s treatment and recommendations for malaria care, including the provision of drug cocktails. They deemed it unnecessary to question or challenge the provider’s advice. This norm persisted even in the absence of a suggested diagnosis from the provider. In this sense, providers are very influential in terms of the decision making process, a finding supported by other research [36,37].

Despite this high regard for providers in Cambodian society, many patients do draw their own conclusions about the cause of their fever, prompting them to sometimes operate proactively with providers. As discovered in other research where malaria is described as an individual disease identifiable from previous symptom episodes [38], many respondents in this study associated their symptoms with previous experiences with the disease. Consequently, they felt confident engaging in self-diagnosis and directly asking for malaria treatment from providers before these practitioners even performed their own exams. These patients are also hesitant to spend additional resources on tests when they “know” they have malaria, a factor that may lead to refusals of diagnostic testing.

The patient-provider interaction also complicates the treatment outcome, because the patients may be misguided by providers or are unclear about the recommended treatment or the results of their diagnostic test. Some study respondents reported being confused about the results of their malaria test, relating how they were given anti-malarials even after testing negative for malaria. Others noted that many providers did not recommend or offer a diagnostic test even when they sought treatment, a finding supported by quantitative provider and household studies [10,18,39]. In one of these studies, one third of the surveyed providers believed that malaria infection could still be confirmed without a blood test, even though the vast majority (88%) understood that other diseases can also cause malaria symptoms [39]. In addition to not recommending diagnostic testing, providers interacting with participants in this current qualitative study also appeared to deliver inappropriate care or improperly prescribed medicines. Such findings are supported by other Cambodian research studies, which found that a large proportion of prescriptions contain two or more drugs that could result in adverse drug reactions, as well as inappropriate practices such as over prescription of medicines, improper instructions on treatment duration and the provision of incorrect medicines and/or their dosages [14].

### Interpreting findings for programmatic decision making

Given the complexity of treatment-seeking behaviour for malaria, the findings from this qualitative study may be useful in shaping communications programs and other interventions that aim to increase informed demand for appropriate management of malaria. The practical recommendations and programmatic considerations that follow suggest ways to change treatment-seeking behaviour as well as improve patient-provider interactions.

#### Incorporate traditional medicines into behaviour change communication messages

The use of traditional medicines is deeply rooted in Cambodian cultural beliefs and norms. Rather than eschewing these remedies, interventions that promote diagnostic testing and first-line treatment for confirmed malaria cases could also consider incorporating local methods just for symptom relief until proper care can be found. For example, communication campaigns could promote the use of specific traditional medicines that are believed to reduce fever for when the patient first experiences symptoms and needs relief while travelling to a facility for a diagnosis and more effective treatment.

#### Employ simple messages in all BCC materials

Given the complexity of treatment-seeking behaviour in Cambodia, clear and simple messaging is needed in all communication materials directed at patients [40,41]. As proposed in a recent review of socially marketed ACT and RDT in Cambodia, some suggested messages include: 1) “If you are going to buy an anti-malarial, only buy the recommended ACT”; 2) “Before you buy an anti-malarial, get tested first”; and 3) “If you test negative, don’t take an anti-malarial” [13]. Existing campaigns that highlight the dangers of cocktails may also want to ensure simple messages address the incorrect perception that cocktails are more effective than pre-packaged medicines, particularly for treating multiple symptoms.

#### Encourage patients to ask for diagnostic testing and appropriate treatments

Even though Cambodians have a high regard for providers and often trust their advice without question, this study and others demonstrate that providers do not always practice appropriate case management of malaria. As such, BCC campaigns should encourage patients to advocate for correct and comprehensive care from their providers. Messages could educate patients about the care they should expect from a health facility, instructing them to ask for diagnostic testing and proper anti-malarial treatments. Findings from this study suggest that Cambodian patients may be open to this messaging approach, given that they currently feel confident self-diagnosing malaria, refusing testing and directly asking a provider for medicine. The intervention goal would be to convince patients to change their “ask” – from cocktail medicines without a confirmed diagnosis to a full course of first-line treatment based on a diagnostic test first.

#### Focus interventions specifically on provider behaviour and education

The importance of provider practices and their influence on the patient are clearly important in determining what the patient receives, as identified in this study as well as through other research [30,42-45]. Such influence is of particular concern when the evidence demonstrates poor provider practices and lack of adherence to Cambodia’s case management strategy. A wide range of interventions can lead to key improvements in professional practice and patient outcomes [42,46,47]. For example, training of practitioners, provider incentives, recurring supervisory visits, clear treatment protocols and a regular supply of equipment are essential for encouraging appropriate malaria diagnosis and treatment [47-49]. Clear guidelines should also be provided to practitioners on how to manage patients presenting with malaria-like symptoms but who are parasite-negative. For designing and monitoring structured and systematic interventions, organisations can employ a number of practical tools, such as provider-based logframes [50], validated behaviour change frameworks and provider behaviour change models [51-54].

#### Emphasize the inappropriateness of drug cocktails in provider education

The positive associations and perceptions of cocktail medicines suggest that behaviour change communication cannot ignore these formulations and leave them out of campaign messaging. As health providers are the ones compiling these cocktail packages, interventions should target this audience through provider education, mentoring and supervision, emphasising the distribution of complete packages of appropriate ACT for confirmed malaria cases.

### Study limitations

As with any qualitative research endeavour, researchers trade off the generalisability of the findings for richness and depth in the data. In this study, the non-random sampling procedure and small sample size reduces the representativeness of the results. Furthermore, the snowball procedure used to recruit participants may have biased the sample, as people are more likely to refer others who are similar to themselves. In addition, the results are specific to the population sampled, given the focus on treatment seeking in just two areas of Cambodia as well as the inclusion of adults reporting malaria fever only. Therefore, the results may not be generalisable to other areas in the country or Southeast Asian region, and they cannot be extrapolated to treatment decisions for other types of fever. Additional research is needed to identify whether these findings are generalisable to a wider population. A final study limitation is that the transcripts were not back translated into English to identify discrepancies.

### Areas for future research

While this qualitative research increases understanding of malaria treatment-seeking behaviour in Cambodia and provides valuable insight into the patient perspective, there still remains a dearth of evidence on the supply side (i.e. the providers) of malaria treatment. Further research is needed on the quality of Cambodian health services, the nature of patient-provider interactions from the provider perspective, and in particular, the array of factors that influence and motivate providers’ testing and dispensing behaviours with regards to malaria. Provider research is also needed in the area of malaria drug cocktails, to understand how attitudes and practices may be changing as well as to pinpoint the types of interventions that may prove successful in combating the widespread misuse of these medicine combinations. Additional research on cocktails should also determine what medicines are found in these mixtures (e.g. anti-malarials, antibiotics) to better understand how these cocktails may be used to treat illnesses other than malaria, a topic currently being investigated by a number of research collaborators [55]. Finally, malaria treatment interventions will benefit from research studies focused on patient costs, access issues and provider financial incentives. While previous research has documented the availability, price, market share and use of anti-malarials in Cambodia [15,17,18], no known studies have linked demand and supply side data to determine Cambodians’ ability to access these first-line treatments.

## Conclusion

Although substantial gains have been made in malaria treatment and service delivery in Cambodia, there is still room for improvement in terms of appropriate case management for suspected malaria cases – namely, higher levels of diagnostic testing prior to treatment and a shift away from the provision of drug cocktails for the disease. Given the pressing issue of artemisinin resistance in the area, including multidrug resistance, encouraging providers to adopt both behaviours is essential.

This study – one of the first of its kind in Cambodia and in Southeast Asia – examines the demand-side factors that influence patients’ behaviour in treatment choice, the sequence of treatment taken and provider interactions, including the acceptance and demand for testing and first-line anti-malarials. Effective intervention programmes will leverage these demand-side factors to promote prompt treatment-seeking behaviour for suspected malaria through channels delivering appropriate case management. On the supply side, given the pivotal role providers play in ensuring the delivery of appropriate care for malaria and their influence in shaping patient treatment-seeking behaviour, interventions designed to improve provider knowledge and the practice of appropriate case management are also needed. Future malaria intervention programmes and research that considers both the patient’s and the provider’s side of the interaction will strengthen appropriate malaria care in Cambodia and, ultimately, lead to reductions in the malaria disease burden.

## Competing interests

The authors declare they have no competing interests.

## Authors’ contributions

KOC designed the study and data collection instruments, as well as drafted the manuscript. PS participated in the design of the study, and carried out data collection and Khmer qualitative analysis. GS was responsible for the analysis and helped to draft the manuscript. SY, HA and ML assisted with the interpretation of the findings and helped to draft the manuscript. All authors read and approved the final manuscript.
